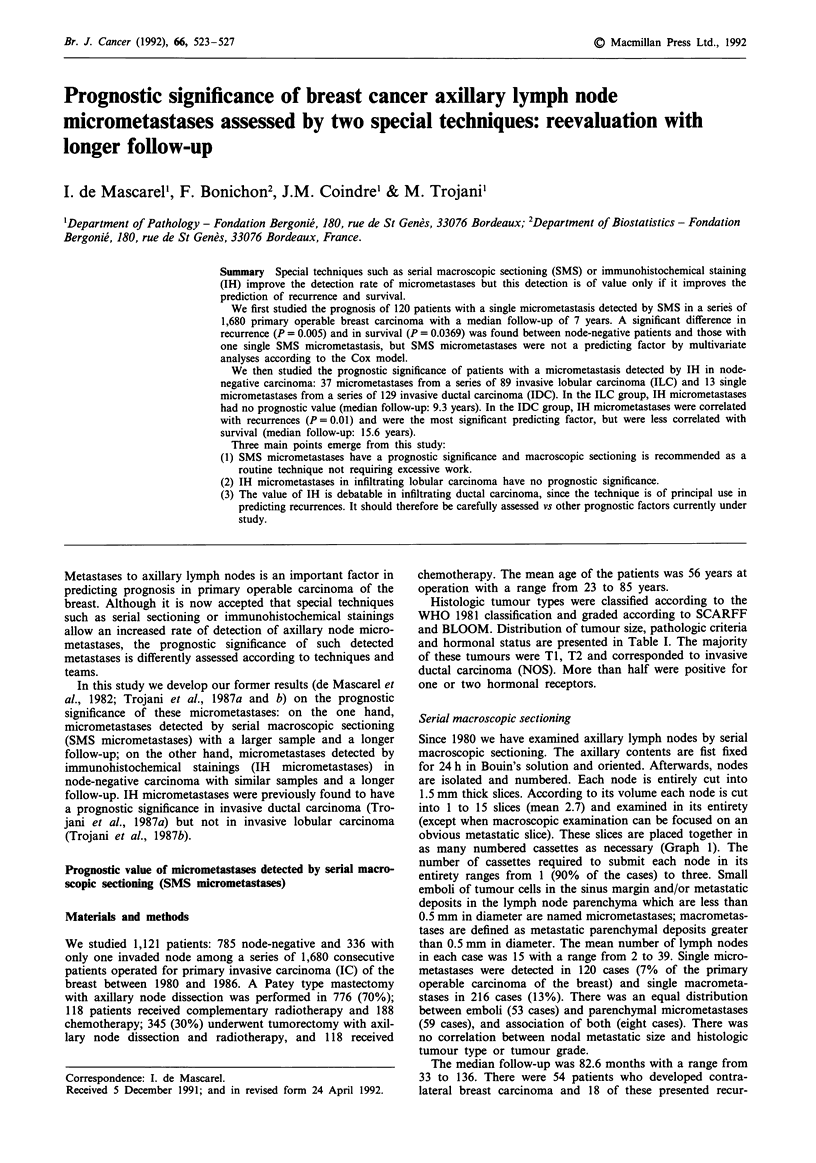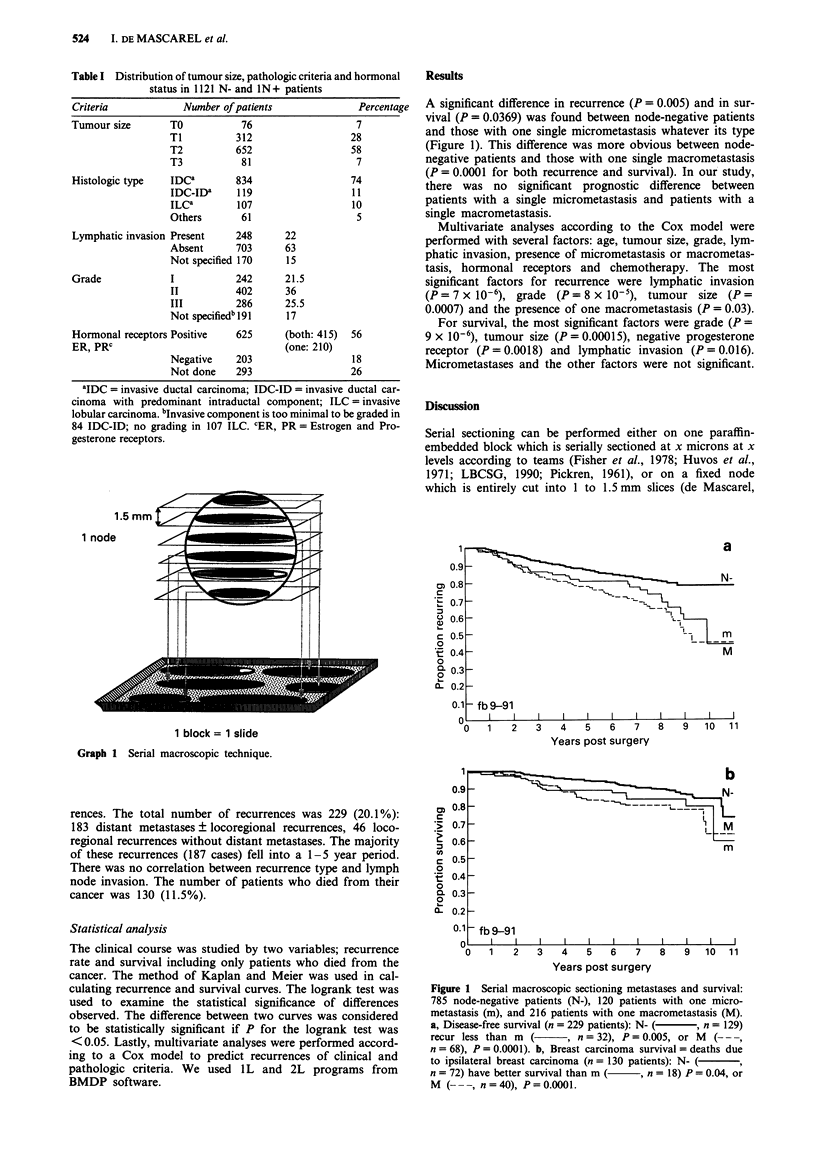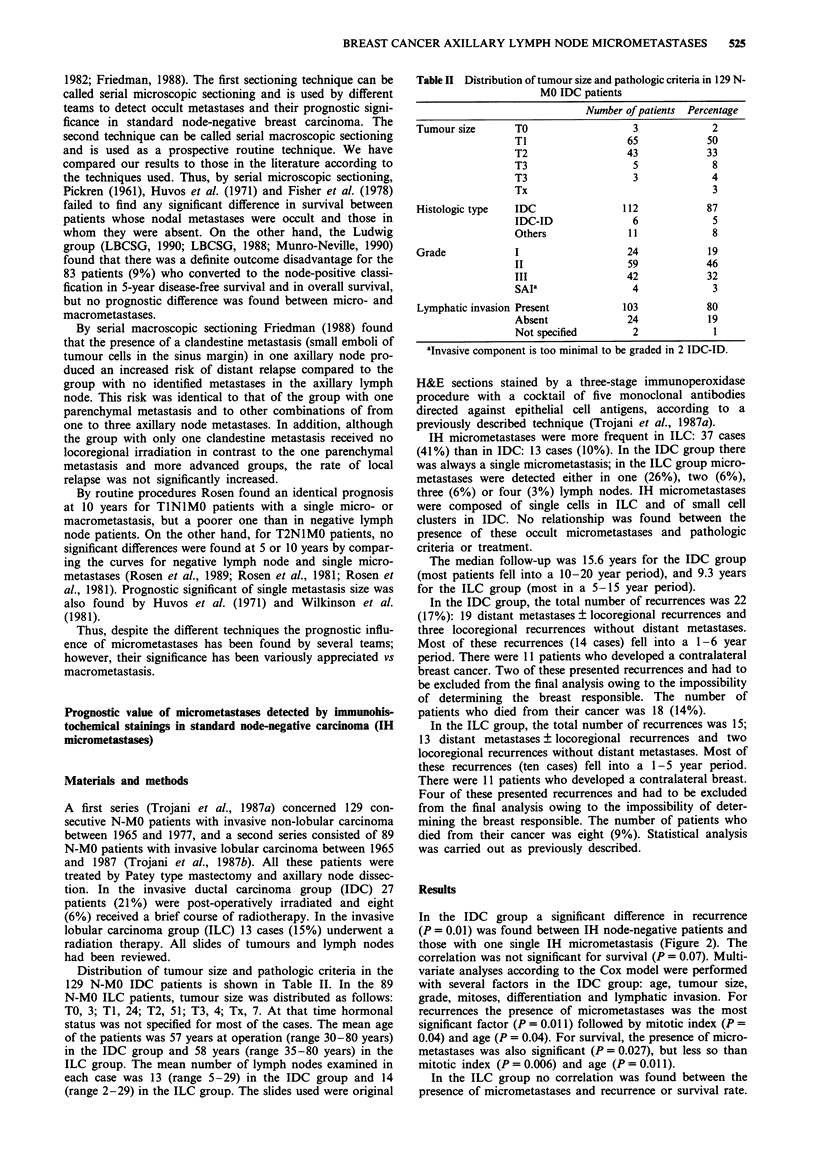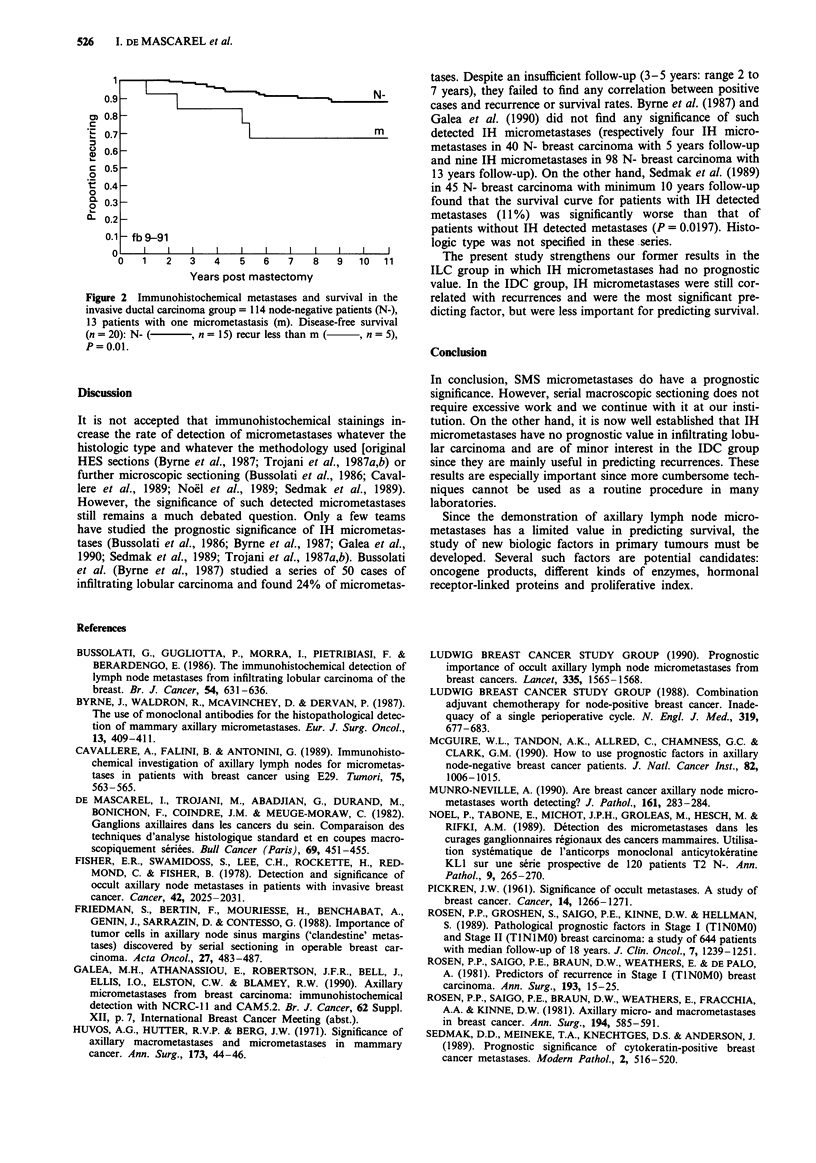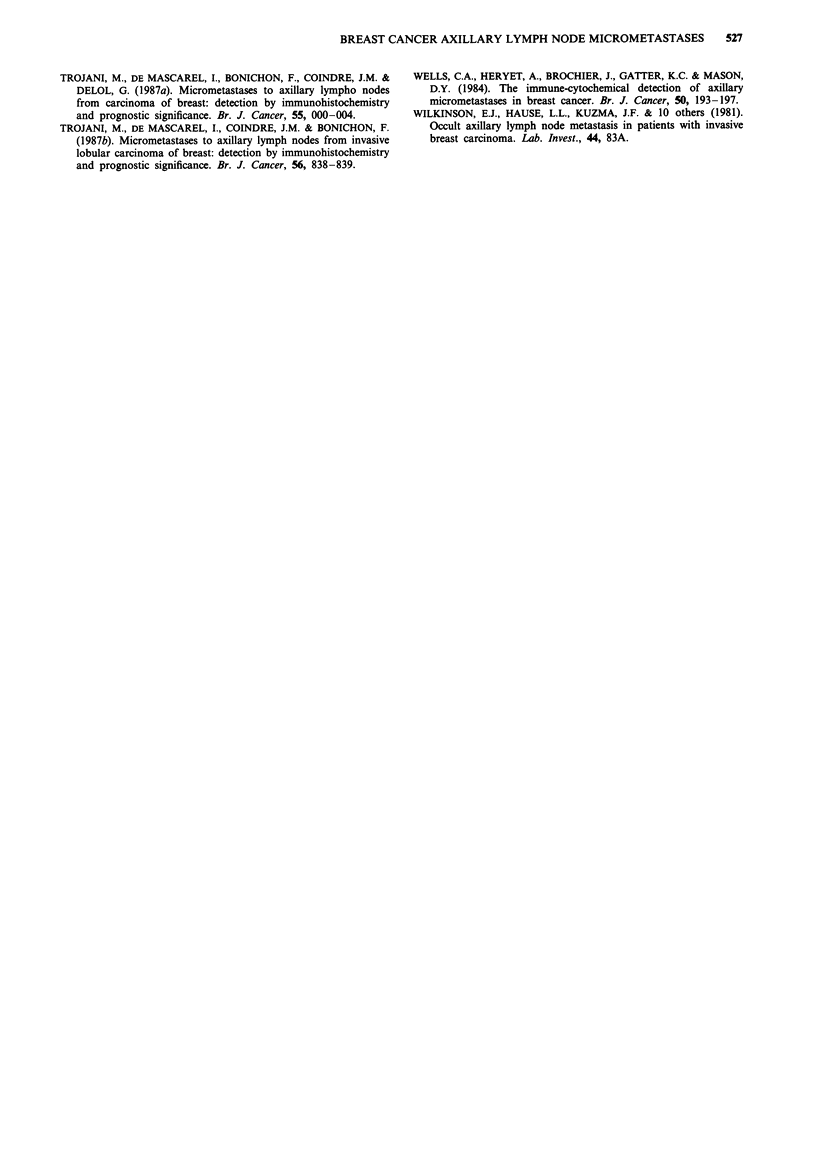# Prognostic significance of breast cancer axillary lymph node micrometastases assessed by two special techniques: reevaluation with longer follow-up.

**DOI:** 10.1038/bjc.1992.306

**Published:** 1992-09

**Authors:** I. de Mascarel, F. Bonichon, J. M. Coindre, M. Trojani

**Affiliations:** Department of Pathology-Fondation Bergonié, Bordeaux, France.

## Abstract

Special techniques such as serial macroscopic sectioning (SMS) or immunohistochemical staining (IH) improve the detection rate of micrometastases but this detection is of value only if it improves the prediction of recurrence and survival. We first studied the prognosis of 120 patients with a single micrometastasis detected by SMS in a series of 1,680 primary operable breast carcinoma with a median follow-up of 7 years. A significant difference in recurrence (P = 0.005) and in survival (P = 0.0369) was found between node-negative patients and those with one single SMS micrometastasis, but SMS micrometastases were not a predicting factor by multivariate analyses according to the Cox model. We then studied the prognostic significance of patients with a micrometastasis detected by IH in node-negative carcinoma: 37 micrometastases from a series of 89 invasive lobular carcinoma (ILC) and 13 single micrometastases from a series of 129 invasive ductal carcinoma (IDC). In the ILC group, IH micrometastases had no prognostic value (median follow-up: 9.3 years). In the IDC group, IH micrometastases were correlated with recurrences (P = 0.01) and were the most significant predicting factor, but were less correlated with survival (median follow-up: 15.6 years). Three main points emerge from this study: (1) SMS micrometastases have a prognostic significance and macroscopic sectioning is recommended as a routine technique not requiring excessive work. (2) IH micrometastases in infiltrating lobular carcinoma have no prognostic significance. (3) The value of IH is debatable in infiltrating ductal carcinoma, since the technique is of principal use in predicting recurrences. It should therefore be carefully assessed vs other prognostic factors currently under study.


Br. J. Cancer (1992), 66, 523–527 © Macmillan Press Ltd., 1992

### Prognostic significance of breast cancer axillary lymph node micrometastases assessed by two special techniques: reevaluation with longer follow-up

I. de Mascarel[1], F. Bonichon[2], J.M. Coindre[1] & M. Trojani[1]

[1]*Department of Pathology – Fondation Bergonié, 180, rue de St Genès, 33076 Bordeaux;* [2]*Department of Biostatistics – Fondation Bergonié, 180, rue de St Genès, 33076 Bordeaux, France.*

**Summary** Special techniques such as serial macroscopic sectioning (SMS) or immunohistochemical staining (IH) improve the detection rate of micrometastases but this detection is of value only if it improves the prediction of recurrence and survival.

We first studied the prognosis of 120 patients with a single micrometastasis detected by SMS in a series of 1,680 primary operable breast carcinoma with a median follow-up of 7 years. A significant difference in recurrence ($P = 0.005$) and in survival ($P = 0.0369$) was found between node-negative patients and those with one single SMS micrometastasis, but SMS micrometastases were not a predicting factor by multivariate analyses according to the Cox model.

We then studied the prognostic significance of patients with a micrometastasis detected by IH in node-negative carcinoma: 37 micrometastases from a series of 89 invasive lobular carcinoma (ILC) and 13 single micrometastases from a series of 129 invasive ductal carcinoma (IDC). In the ILC group, IH micrometastases had no prognostic value (median follow-up: 9.3 years). In the IDC group, IH micrometastases were correlated with recurrences ($P = 0.01$) and were the most significant predicting factor, but were less correlated with survival (median follow-up: 15.6 years).

Three main points emerge from this study:

(1) SMS micrometastases have a prognostic significance and macroscopic sectioning is recommended as a routine technique not requiring excessive work.
(2) IH micrometastases in infiltrating lobular carcinoma have no prognostic significance.
(3) The value of IH is debatable in infiltrating ductal carcinoma, since the technique is of principal use in predicting recurrences. It should therefore be carefully assessed *vs* other prognostic factors currently under study.

Metastases to axillary lymph nodes is an important factor in predicting prognosis in primary operable carcinoma of the breast. Although it is now accepted that special techniques such as serial sectioning or immunohistochemical stainings allow an increased rate of detection of axillary node micrometastases, the prognostic significance of such detected metastases is differently assessed according to techniques and teams.

In this study we develop our former results (de Mascarel *et al.*, 1982; Trojani *et al.*, 1987*a* and *b*) on the prognostic significance of these micrometastases: on the one hand, micrometastases detected by serial macroscopic sectioning (SMS micrometastases) with a larger sample and a longer follow-up; on the other hand, micrometastases detected by immunohistochemical stainings (IH micrometastases) in node-negative carcinoma with similar samples and a longer follow-up. IH micrometastases were previously found to have a prognostic significance in invasive ductal carcinoma (Trojani *et al.*, 1987*a*) but not in invasive lobular carcinoma (Trojani *et al.*, 1987*b*).

### Prognostic value of micrometastases detected by serial macroscopic sectioning (SMS micrometastases)

### Materials and methods

We studied 1,121 patients: 785 node-negative and 336 with only one invaded node among a series of 1,680 consecutive patients operated for primary invasive carcinoma (IC) of the breast between 1980 and 1986. A Patey type mastectomy with axillary node dissection was performed in 776 (70%); 118 patients received complementary radiotherapy and 188 chemotherapy; 345 (30%) underwent tumorectomy with axillary node dissection and radiotherapy, and 118 received chemotherapy. The mean age of the patients was 56 years at operation with a range from 23 to 85 years.

Histologic tumour types were classified according to the WHO 1981 classification and graded according to SCARFF and BLOOM. Distribution of tumour size, pathologic criteria and hormonal status are presented in Table I. The majority of these tumours were T1, T2 and corresponded to invasive ductal carcinoma (NOS). More than half were positive for one or two hormonal receptors.

#### *Serial macroscopic sectioning*

Since 1980 we have examined axillary lymph nodes by serial macroscopic sectioning. The axillary contents are fist fixed for 24 h in Bouin's solution and oriented. Afterwards, nodes are isolated and numbered. Each node is entirely cut into 1.5 mm thick slices. According to its volume each node is cut into 1 to 15 slices (mean 2.7) and examined in its entirety (except when macroscopic examination can be focused on an obvious metastatic slice). These slices are placed together in as many numbered cassettes as necessary (Graph 1). The number of cassettes required to submit each node in its entirety ranges from 1 (90% of the cases) to three. Small emboli of tumour cells in the sinus margin and/or metastatic deposits in the lymph node parenchyma which are less than 0.5 mm in diameter are named micrometastases; macrometastases are defined as metastatic parenchymal deposits greater than 0.5 mm in diameter. The mean number of lymph nodes in each case was 15 with a range from 2 to 39. Single micrometastases were detected in 120 cases (7% of the primary operable carcinoma of the breast) and single macrometastases in 216 cases (13%). There was an equal distribution between emboli (53 cases) and parenchymal micrometastases (59 cases), and association of both (eight cases). There was no correlation between nodal metastatic size and histologic tumour type or tumour grade.

The median follow-up was 82.6 months with a range from 33 to 136. There were 54 patients who developed contralateral breast carcinoma and 18 of these presented recur-

Correspondence: I. de Mascarel.
Received 5 December 1991; and in revised form 24 April 1992.

**Table I** Distribution of tumour size, pathologic criteria and hormonal status in 1121 N- and 1N+ patients

| *Criteria* | *Number of patients* | | | *Percentage* |
|---|---|---|---|---|
| Tumour size | T0 | 76 | | 7 |
| | T1 | 312 | | 28 |
| | T2 | 652 | | 58 |
| | T3 | 81 | | 7 |
| Histologic type | IDC[a] | 834 | | 74 |
| | IDC-ID[a] | 119 | | 11 |
| | ILC[a] | 107 | | 10 |
| | Others | 61 | | 5 |
| Lymphatic invasion | Present | 248 | 22 | |
| | Absent | 703 | 63 | |
| | Not specified | 170 | 15 | |
| Grade | I | 242 | 21.5 | |
| | II | 402 | 36 | |
| | III | 286 | 25.5 | |
| | Not specified[b] | 191 | 17 | |
| Hormonal receptors ER, PR[c] | Positive | 625 | (both: 415) (one: 210) | 56 |
| | Negative | 203 | | 18 |
| | Not done | 293 | | 26 |

[a]IDC = invasive ductal carcinoma; IDC-ID = invasive ductal carcinoma with predominant intraductal component; ILC = invasive lobular carcinoma. [b]Invasive component is too minimal to be graded in 84 IDC-ID; no grading in 107 ILC. [c]ER, PR = Estrogen and Progesterone receptors.

**Graph 1** Serial macroscopic technique.

rences. The total number of recurrences was 229 (20.1%): 183 distant metastases ± locoregional recurrences, 46 locoregional recurrences without distant metastases. The majority of these recurrences (187 cases) fell into a 1–5 year period. There was no correlation between recurrence type and lymph node invasion. The number of patients who died from their cancer was 130 (11.5%).

### *Statistical analysis*

The clinical course was studied by two variables; recurrence rate and survival including only patients who died from the cancer. The method of Kaplan and Meier was used in calculating recurrence and survival curves. The logrank test was used to examine the statistical significance of differences observed. The difference between two curves was considered to be statistically significant if $P$ for the logrank test was $<0.05$. Lastly, multivariate analyses were performed according to a Cox model to predict recurrences of clinical and pathologic criteria. We used 1L and 2L programs from BMDP software.

### Results

A significant difference in recurrence ($P = 0.005$) and in survival ($P = 0.0369$) was found between node-negative patients and those with one single micrometastasis whatever its type (Figure 1). This difference was more obvious between node-negative patients and those with one single macrometastasis ($P = 0.0001$ for both recurrence and survival). In our study, there was no significant prognostic difference between patients with a single micrometastasis and patients with a single macrometastasis.

Multivariate analyses according to the Cox model were performed with several factors: age, tumour size, grade, lymphatic invasion, presence of micrometastasis or macrometastasis, hormonal receptors and chemotherapy. The most significant factors for recurrence were lymphatic invasion ($P = 7 \times 10^{-6}$), grade ($P = 8 \times 10^{-5}$), tumour size ($P = 0.0007$) and the presence of one macrometastasis ($P = 0.03$).

For survival, the most significant factors were grade ($P = 9 \times 10^{-6}$), tumour size ($P = 0.00015$), negative progesterone receptor ($P = 0.0018$) and lymphatic invasion ($P = 0.016$). Micrometastases and the other factors were not significant.

### Discussion

Serial sectioning can be performed either on one paraffin-embedded block which is serially sectioned at $x$ microns at $x$ levels according to teams (Fisher *et al.*, 1978; Huvos *et al.*, 1971; LBCSG, 1990; Pickren, 1961), or on a fixed node which is entirely cut into 1 to 1.5 mm slices (de Mascarel,

**Figure 1** Serial macroscopic sectioning metastases and survival: 785 node-negative patients (N-), 120 patients with one micrometastasis (m), and 216 patients with one macrometastasis (M). **a**, Disease-free survival ($n = 229$ patients): N- (———, $n = 129$) recur less than m (———, $n = 32$), $P = 0.005$, or M (– – –, $n = 68$), $P = 0.0001$). **b**, Breast carcinoma survival = deaths due to ipsilateral breast carcinoma ($n = 130$ patients): N- (———, $n = 72$) have better survival than m (———, $n = 18$) $P = 0.04$, or M (– – –, $n = 40$), $P = 0.0001$.

1982; Friedman, 1988). The first sectioning technique can be called serial microscopic sectioning and is used by different teams to detect occult metastases and their prognostic significance in standard node-negative breast carcinoma. The second technique can be called serial macroscopic sectioning and is used as a prospective routine technique. We have compared our results to those in the literature according to the techniques used. Thus, by serial microscopic sectioning, Pickren (1961), Huvos *et al.* (1971) and Fisher *et al.* (1978) failed to find any significant difference in survival between patients whose nodal metastases were occult and those in whom they were absent. On the other hand, the Ludwig group (LBCSG, 1990; LBCSG, 1988; Munro-Neville, 1990) found that there was a definite outcome disadvantage for the 83 patients (9%) who converted to the node-positive classification in 5-year disease-free survival and in overall survival, but no prognostic difference was found between micro- and macrometastases.

By serial macroscopic sectioning Friedman (1988) found that the presence of a clandestine metastasis (small emboli of tumour cells in the sinus margin) in one axillary node produced an increased risk of distant relapse compared to the group with no identified metastases in the axillary lymph node. This risk was identical to that of the group with one parenchymal metastasis and to other combinations of from one to three axillary node metastases. In addition, although the group with only one clandestine metastasis received no locoregional irradiation in contrast to the one parenchymal metastasis and more advanced groups, the rate of local relapse was not significantly increased.

By routine procedures Rosen found an identical prognosis at 10 years for T1N1M0 patients with a single micro- or macrometastasis, but a poorer one than in negative lymph node patients. On the other hand, for T2N1M0 patients, no significant differences were found at 5 or 10 years by comparing the curves for negative lymph node and single micrometastases (Rosen *et al.*, 1989; Rosen *et al.*, 1981; Rosen *et al.*, 1981). Prognostic significant of single metastasis size was also found by Huvos *et al.* (1971) and Wilkinson *et al.* (1981).

Thus, despite the different techniques the prognostic influence of micrometastases has been found by several teams; however, their significance has been variously appreciated *vs* macrometastasis.

### Prognostic value of micrometastases detected by immunohistochemical stainings in standard node-negative carcinoma (IH micrometastases)

### Materials and methods

A first series (Trojani *et al.*, 1987*a*) concerned 129 consecutive N-M0 patients with invasive non-lobular carcinoma between 1965 and 1977, and a second series consisted of 89 N-M0 patients with invasive lobular carcinoma between 1965 and 1987 (Trojani *et al.*, 1987*b*). All these patients were treated by Patey type mastectomy and axillary node dissection. In the invasive ductal carcinoma group (IDC) 27 patients (21%) were post-operatively irradiated and eight (6%) received a brief course of radiotherapy. In the invasive lobular carcinoma group (ILC) 13 cases (15%) underwent a radiation therapy. All slides of tumours and lymph nodes had been reviewed.

Distribution of tumour size and pathologic criteria in the 129 N-M0 IDC patients is shown in Table II. In the 89 N-M0 ILC patients, tumour size was distributed as follows: T0, 3; T1, 24; T2, 51; T3, 4; Tx, 7. At that time hormonal status was not specified for most of the cases. The mean age of the patients was 57 years at operation (range 30–80 years) in the IDC group and 58 years (range 35–80 years) in the ILC group. The mean number of lymph nodes examined in each case was 13 (range 5–29) in the IDC group and 14 (range 2–29) in the ILC group. The slides used were original H&E sections stained by a three-stage immunoperoxidase procedure with a cocktail of five monoclonal antibodies directed against epithelial cell antigens, according to a previously described technique (Trojani *et al.*, 1987*a*).

**Table II** Distribution of tumour size and pathologic criteria in 129 N-M0 IDC patients

| | | *Number of patients* | *Percentage* |
|---|---|---|---|
| Tumour size | T0 | 3 | 2 |
| | T1 | 65 | 50 |
| | T2 | 43 | 33 |
| | T3 | 5 | 8 |
| | T3 | 3 | 4 |
| | Tx | | 3 |
| Histologic type | IDC | 112 | 87 |
| | IDC-ID | 6 | 5 |
| | Others | 11 | 8 |
| Grade | I | 24 | 19 |
| | II | 59 | 46 |
| | III | 42 | 32 |
| | SAI[a] | 4 | 3 |
| Lymphatic invasion | Present | 103 | 80 |
| | Absent | 24 | 19 |
| | Not specified | 2 | 1 |

[a]Invasive component is too minimal to be graded in 2 IDC-ID.

IH micrometastases were more frequent in ILC: 37 cases (41%) than in IDC: 13 cases (10%). In the IDC group there was always a single micrometastasis; in the ILC group micrometastases were detected either in one (26%), two (6%), three (6%) or four (3%) lymph nodes. IH micrometastases were composed of single cells in ILC and of small cell clusters in IDC. No relationship was found between the presence of these occult micrometastases and pathologic criteria or treatment.

The median follow-up was 15.6 years for the IDC group (most patients fell into a 10–20 year period), and 9.3 years for the ILC group (most in a 5–15 year period).

In the IDC group, the total number of recurrences was 22 (17%): 19 distant metastases ± locoregional recurrences and three locoregional recurrences without distant metastases. Most of these recurrences (14 cases) fell into a 1–6 year period. There were 11 patients who developed a contralateral breast cancer. Two of these presented recurrences and had to be excluded from the final analysis owing to the impossibility of determining the breast responsible. The number of patients who died from their cancer was 18 (14%).

In the ILC group, the total number of recurrences was 15; 13 distant metastases ± locoregional recurrences and two locoregional recurrences without distant metastases. Most of these recurrences (ten cases) fell into a 1–5 year period. There were 11 patients who developed a contralateral breast. Four of these presented recurrences and had to be excluded from the final analysis owing to the impossibility of determining the breast responsible. The number of patients who died from their cancer was eight (9%). Statistical analysis was carried out as previously described.

### Results

In the IDC group a significant difference in recurrence ($P = 0.01$) was found between IH node-negative patients and those with one single IH micrometastasis (Figure 2). The correlation was not significant for survival ($P = 0.07$). Multivariate analyses according to the Cox model were performed with several factors in the IDC group: age, tumour size, grade, mitoses, differentiation and lymphatic invasion. For recurrences the presence of micrometastases was the most significant factor ($P = 0.011$) followed by mitotic index ($P = 0.04$) and age ($P = 0.04$). For survival, the presence of micrometastases was also significant ($P = 0.027$), but less so than mitotic index ($P = 0.006$) and age ($P = 0.011$).

In the ILC group no correlation was found between the presence of micrometastases and recurrence or survival rate.

**Figure 2** Immunohistochemical metastases and survival in the invasive ductal carcinoma group = 114 node-negative patients (N-), 13 patients with one micrometastasis (m). Disease-free survival ($n = 20$): N- (———, $n = 15$) recur less than m (———, $n = 5$), $P = 0.01$.

### Discussion

It is not accepted that immunohistochemical stainings increase the rate of detection of micrometastases whatever the histologic type and whatever the methodology used [original HES sections (Byrne *et al.*, 1987; Trojani *et al.*, 1987*a*,*b*) or further microscopic sectioning (Bussolati *et al.*, 1986; Cavallere *et al.*, 1989; Noël *et al.*, 1989; Sedmak *et al.*, 1989). However, the significance of such detected micrometastases still remains a much debated question. Only a few teams have studied the prognostic significance of IH micrometastases (Bussolati *et al.*, 1986; Byrne *et al.*, 1987; Galea *et al.*, 1990; Sedmak *et al.*, 1989; Trojani *et al.*, 1987*a*,*b*). Bussolati *et al.* (Byrne *et al.*, 1987) studied a series of 50 cases of infiltrating lobular carcinoma and found 24% of micrometastases. Despite an insufficient follow-up (3–5 years: range 2 to 7 years), they failed to find any correlation between positive cases and recurrence or survival rates. Byrne *et al.* (1987) and Galea *et al.* (1990) did not find any significance of such detected IH micrometastases (respectively four IH micrometastases in 40 N- breast carcinoma with 5 years follow-up and nine IH micrometastases in 98 N- breast carcinoma with 13 years follow-up). On the other hand, Sedmak *et al.* (1989) in 45 N- breast carcinoma with minimum 10 years follow-up found that the survival curve for patients with IH detected metastases (11%) was significantly worse than that of patients without IH detected metastases ($P = 0.0197$). Histologic type was not specified in these series.

The present study strengthens our former results in the ILC group in which IH micrometastases had no prognostic value. In the IDC group, IH micrometastases were still correlated with recurrences and were the most significant predicting factor, but were less important for predicting survival.

### Conclusion

In conclusion, SMS micrometastases do have a prognostic significance. However, serial macroscopic sectioning does not require excessive work and we continue with it at our institution. On the other hand, it is now well established that IH micrometastases have no prognostic value in infiltrating lobular carcinoma and are of minor interest in the IDC group since they are mainly useful in predicting recurrences. These results are especially important since more cumbersome techniques cannot be used as a routine procedure in many laboratories.

Since the demonstration of axillary lymph node micrometastases has a limited value in predicting survival, the study of new biologic factors in primary tumours must be developed. Several such factors are potential candidates: oncogene products, different kinds of enzymes, hormonal receptor-linked proteins and proliferative index.

### References

BUSSOLATI, G., GUGLIOTTA, P., MORRA, I., PIETRIBIASI, F. & BERARDENGO, E. (1986). The immunohistochemical detection of lymph node metastases from infiltrating lobular carcinoma of the breast. *Br. J. Cancer*, **54**, 631–636.

BYRNE, J., WALDRON, R., MCAVINCHEY, D. & DERVAN, P. (1987). The use of monoclonal antibodies for the histopathological detection of mammary axillary micrometastases. *Eur. J. Surg. Oncol.*, **13**, 409–411.

CAVALLERE, A., FALINI, B. & ANTONINI, G. (1989). Immunohistochemical investigation of axillary lymph nodes for micrometastases in patients with breast cancer using E29. *Tumori*, **75**, 563–565.

DE MASCAREL, I., TROJANI, M., ABADJIAN, G., DURAND, M., BONICHON, F., COINDRE, J.M. & MEUGE-MORAW, C. (1982). Ganglions axillaires dans les cancers du sein. Comparaison des techniques d'analyse histologique standard et en coupes macroscopiquement sériées. *Bull Cancer (Paris)*, **69**, 451–455.

FISHER, E.R., SWAMIDOSS, S., LEE, C.H., ROCKETTE, H., REDMOND, C. & FISHER, B. (1978). Detection and significance of occult axillary node metastases in patients with invasive breast cancer. *Cancer*, **42**, 2025–2031.

FRIEDMAN, S., BERTIN, F., MOURIESSE, H., BENCHABAT, A., GENIN, J., SARRAZIN, D. & CONTESSO, G. (1988). Importance of tumor cells in axillary node sinus margins ('clandestine' metastases) discovered by serial sectioning in operable breast carcinoma. *Acta Oncol.*, **27**, 483–487.

GALEA, M.H., ATHANASSIOU, E., ROBERTSON, J.F.R., BELL, J., ELLIS, I.O., ELSTON, C.W. & BLAMEY, R.W. (1990). Axillary micrometastases from breast carcinoma: immunohistochemical detection with NCRC-11 and CAM5.2. *Br. J. Cancer*, **62** Suppl. XII, p. 7, International Breast Cancer Meeting (abst.).

HUVOS, A.G., HUTTER, R.V.P. & BERG, J.W. (1971). Significance of axillary macrometastases and micrometastases in mammary cancer. *Ann. Surg.*, **173**, 44–46.

LUDWIG BREAST CANCER STUDY GROUP (1990). Prognostic importance of occult axillary lymph node micrometastases from breast cancers. *Lancet*, **335**, 1565–1568.

LUDWIG BREAST CANCER STUDY GROUP (1988). Combination adjuvant chemotherapy for node-positive breast cancer. Inadequacy of a single perioperative cycle. *N. Engl. J. Med.*, **319**, 677–683.

MCGUIRE, W.L., TANDON, A.K., ALLRED, C., CHAMNESS, G.C. & CLARK, G.M. (1990). How to use prognostic factors in axillary node-negative breast cancer patients. *J. Natl. Cancer Inst.*, **82**, 1006–1015.

MUNRO-NEVILLE, A. (1990). Are breast cancer axillary node micrometastases worth detecting? *J. Pathol.*, **161**, 283–284.

NOEL, P., TABONE, E., MICHOT, J.P.H., GROLEAS, M., HESCH, M. & RIFKI, A.M. (1989). Détection des micrometastases dans les curages ganglionnaires régionaux des cancers mammaires. Utilisation systématique de l'anticorps monoclonal anticytokératine KL1 sur une série prospective de 120 patients T2 N-. *Ann. Pathol.*, **9**, 265–270.

PICKREN, J.W. (1961). Significance of occult metastases. A study of breast cancer. *Cancer*, **14**, 1266–1271.

ROSEN, P.P., GROSHEN, S., SAIGO, P.E., KINNE, D.W. & HELLMAN, S. (1989). Pathological prognostic factors in Stage I (T1N0M0) and Stage II (T1N1M0) breast carcinoma: a study of 644 patients with median follow-up of 18 years. *J. Clin. Oncol.*, **7**, 1239–1251.

ROSEN, P.P., SAIGO, P.E., BRAUN, D.W., WEATHERS, E. & DE PALO, A. (1981). Predictors of recurrence in Stage I (T1N0M0) breast carcinoma. *Ann. Surg.*, **193**, 15–25.

ROSEN, P.P., SAIGO, P.E., BRAUN, D.W., WEATHERS, E., FRACCHIA, A.A. & KINNE, D.W. (1981). Axillary micro- and macrometastases in breast cancer. *Ann. Surg.*, **194**, 585–591.

SEDMAK, D.D., MEINEKE, T.A., KNECHTGES, D.S. & ANDERSON, J. (1989). Prognostic significance of cytokeratin-positive breast cancer metastases. *Modern Pathol.*, **2**, 516–520.

TROJANI, M., DE MASCAREL, I., BONICHON, F., COINDRE, J.M. & DELOL, G. (1987*a*). Micrometastases to axillary lympho nodes from carcinoma of breast: detection by immunohistochemistry and prognostic significance. *Br. J. Cancer*, **55**, 000–004.

TROJANI, M., DE MASCAREL, I., COINDRE, J.M. & BONICHON, F. (1987*b*). Micrometastases to axillary lymph nodes from invasive lobular carcinoma of breast: detection by immunohistochemistry and prognostic significance. *Br. J. Cancer*, **56**, 838–839.

WELLS, C.A., HERYET, A., BROCHIER, J., GATTER, K.C. & MASON, D.Y. (1984). The immune-cytochemical detection of axillary micrometastases in breast cancer. *Br. J. Cancer*, **50**, 193–197.

WILKINSON, E.J., HAUSE, L.L., KUZMA, J.F. & 10 others (1981). Occult axillary lymph node metastasis in patients with invasive breast carcinoma. *Lab. Invest.*, **44**, 83A.